# Widening Excess Mortality During the COVID-19 Pandemic in Individuals Who Self-Harmed: A Whole-Population-Based E-Cohort Study in Wales, UK, April 2016–March 2021

**DOI:** 10.1027/0227-5910/a000882

**Published:** 2022-10-13

**Authors:** Sze Chim Lee, Marcos DelPozo-Banos, Yasmin Friedmann, Ashley Akbari, Ronan A. Lyons, Ann John

**Affiliations:** ^1^ Population Data Science, Swansea University Medical School, Swansea, UK

**Keywords:** COVID-19, death, electronic health records, mortality, self-harm

## Abstract

**Abstract:** *Background:* Studies on COVID-19 pandemic-associated changes in mortality following self-harm remain scarce and inconclusive. *Aims:* To compare mortality risks in individuals who had self-harmed to those for individuals who had not, before and during the COVID-19 pandemic (Waves 1 and 2) in Wales, the United Kingdom, using population-based routinely collected data. *Method:* We linked whole population health data to all-cause mortality following an episode of self-harm between April 2016 and March 2021. Propensity score matching, Cox regression, and difference-in-differences were applied to compute changes in excess mortality (as ratios of hazard ratios, RHRs) before and during the pandemic for individuals who self-harmed. *Results:* The difference in mortality for individuals who self-harmed compared to those who did not widened during Wave 1 (RHR = 2.03, 95% CI: 1.04–4.03) and Wave 2 (RHR = 2.19, 95% CI: 1.12–4.29) from before the pandemic. Stratification by sex and age group produced no significant subgroup differences although risk for younger than 65 years group were higher. *Limitations:* Limitations include small sample size and incomplete data on cause-specific deaths during the pandemic. *Conclusion:* Our results underscore continuous monitoring of mortality of individuals who self-harm and effective interventions to address any increases in mortality.

The impact of the COVID-19 pandemic on mortality has been under close scrutiny (Beaney et al., 2020). While some studies reported elevated COVID-19 mortality for at-risk individuals, (e.g., those with mental health conditions; Toubasi et al., 2021), others advocated evaluating total lives lost to capture potential detrimental effects associated with mitigations to curb its spread (VanderWeele, 2020). Mortality for individuals who self-harmed was higher than for the general population prior to the pandemic (Bergen et al., 2012). Data on change in mortality during the pandemic for this subpopulation are scarce. A recent study reported increased mortality for individuals hospitalized for self-harm during the pandemic in France (Jollant et al., 2021). However, the findings were limited by using only hospitalization data covering the early months of the pandemic and did not consider changes in mortality in the general population.

This study aimed to examine any changes in mortality difference before and during the COVID-19 pandemic for individuals who self-harmed compared to those who did not.

## Methods

### Design and Study Population

This e-cohort study used anonymized individual-level population-based routinely collected linkable data in Wales, the United Kingdom, from April 2016 to March 2021, the study period (Figure E1a in Electronic Supplementary Material 1 [ESM 1] and RECORD checklist in ESM 2). Data sources were accessed through the *Controlling COVID-19* cohort within the Secure Anonymised Information Linkage (SAIL) Databank, a multisourced repository holding anonymized data for the ∼3.5 million population of Wales (Lyons et al., 2020). SAIL’s Information Governance Review Panel granted ethical approval (Project 0911). Data sources are listed in Table E1 in ESM 1, and data linkage between SAIL data sources were outlined in the methods document in ESM 3.

We included individuals who lived in Wales for at least 1 month within the study period (Figure E1b in ESM 1). We defined the *self-harm* group as individuals presenting to healthcare services with self-harm during the study period and the *no self-harm* group as those without self-harm event based on available records. We defined the index date as the date of first self-harm for the self-harm group and a randomized date conditional on the distribution of the index date of the self-harm group for the no self-harm group (Figure E2 in ESM 1) and only considered individuals aged 10 years or older at the index date.

### Measures

**Figure 1:**
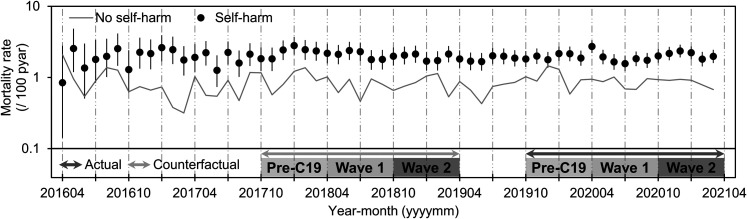
Monthly trends (in log scale) of crude all-cause mortality rates for self-harm and no self-harm group from the matched self-harm cohort between April 2016 and March 2021. Defined periods for this study: Pre-C19: October 2019–March 2020, Wave 1: April 2020–September 2020, Wave 2: October 2020–March 2021, actual COVID-19 period: 2019–2021, and counterfactual period: 2017–2019. Error bars: 95% CIs. pyar = person-years at risk.

Mortality data were extracted from the Office for National Statistics death register and the Welsh Demographic Service data set (Table E1 in ESM 1). ICD-10 codes were used to group underlying causes of death into natural, unnatural (including suicide as a separate category), and unknown causes (John et al., 2018). Self-harm was identified from primary care, emergency department, and hospital admissions data using validated code lists (Marchant et al., 2020). We defined periods as Pre-C19 (October 2019–March 2020), Wave 1 (April 2020–September 2020), and Wave 2 (October 2020–March 2021; Figure 1). We extracted other covariates (e.g., sex and age) to analyze change in mortality (complete list in Table E2 in ESM 1).

### Statistical Analysis

Statistical analyses are outlined below with details in the methods document in ESM 3.

We calculated crude monthly mortality rates in the self-harm cohort. Due to data quality issues for cause-specific mortality, we have only reported trends of crude mortality rates for self-harm and no self-harm groups as descriptive statistics. For all-cause mortality, we performed one-to-one propensity score matching (PSM) on the self-harm cohort with 6-month (beginning from April to October) time-stratified Cox regression on the matched cohort to compute hazard ratios (HRs) for mortality risk in the self-harm group. We calculated the ratios of HRs (RHRs) by difference-in-difference (DiD) to assess changes in HRs between the Pre-C19 and Wave 1/2 to the counterfactual period (Figure 1). RHRs >1 reflect an increased mortality gap between the self-harm and no self-harm groups during Wave 1 (or 2) compared to the respective changes in mortality gap measured at the counterfactual period. Stratified analyses were conducted by sex, age group, and area deprivation. We conducted robustness checks and two sensitivity analyses: one using incident self-harm population only and the other using the whole population (unmatched) cohort. We also compared the proportion of all-cause mortality for individuals who self-harmed during the pandemic (Wave 1 and 2) with those who self-harmed in the prepandemic period (Pre-C19) using the DiD approach.

## Results

Among the 2,932,232 eligible individuals, we identified 45,422 and 2,558,599 individuals in the self-harm and no self-harm groups with 2,244 and 83,529 deaths, respectively (Table E3 and Figure E1B in ESM 1, see also Figure E3 in ESM 1 for the monthly trend of self-harm within the study period). We observed two peaks in monthly trends of crude rates of all-cause and natural-cause mortality associated with Wave 1 and Wave 2 for both self-harm and no self-harm groups (Figure E4 in ESM 1). After April 2020, we found a decreasing trend of unnatural-causes and suicide mortality rates, at the same time as an increasing trend of unknown-causes mortality rate. The decreases in mortality for both unnatural causes and suicide during the pandemic were more pronounced in the self-harm group.

After PSM (Table E3, Table E4, and Figure E5 in ESM 1, Results in ESM 3), 43,368 individuals from the self-harm group (95.5% of 45,422) were matched with the same number from the no self-harm group. Monthly trends of crude all-cause mortality rates showed peaks corresponding to Waves 1 and 2 for the self-harm group but not for the no self-harm group (Figure 1). RHRs were significantly greater than one for Wave 1 (RHR = 2.03, 95% CI: 1.04–4.03, *p = .*042) and Wave 2 (RHR = 2.19, 95% CI: 1.12–4.29, *p = .*023). Excluding the COVID-19 infection variable from the model revealed similar RHRs, and stratified analyses did not indicate significant subgroup differences although RHR was considerably higher for younger than 65 years (RHR = 3.85) than the older than 65 years (RHR = 1.75) age group in Wave 2 (Table E5, Table E6, and Figure E6 in ESM 1). RHRs from the robustness check were close to unity whereas RHRs from the sensitivity analysis that ascertained only the incident self-harm population to the self-harm group were still greater, but not significantly different from one (Table E5 in ESM 1). Without applying PSM (unmatched), RHRs were slightly reduced compared to the main analysis but were still statistically greater than one. The proportion of mortality for individuals who self-harmed during Wave 1 and Wave 2 was not significantly different from those who self-harmed in the prepandemic period (Table E7a and Table E7b in ESM 1).

## Discussion

We, for the first time, observed a widening mortality gap between individuals who self-harmed and the general population over the COVID-19 pandemic between April 2020 and March 2021 in the United Kingdom. A French analysis showed an elevated all-cause mortality for individuals hospitalized for self-harm during the early months of the pandemic (Jollant et al., 2021). We employed PSM and DiD to balance characteristics between the self-harm and general population and account for baseline mortality risk following self-harm before the pandemic. However, correct model specification of propensity scores and no unmeasured confounding assumptions for PSM and DiD may not be easily verified. The negative findings from the sensitivity analysis using incident self-harm population may stem from smaller sample size and difference in time since first exposure to self-harm comparing incident to prevalent samples (Vandenbroucke & Pearce, 2015). Caution is required in interpreting our findings as longer-term consequences associated with COVID-19 (e.g., possible economic downturn; VanderWeele, 2020), were not captured. We found similar trends in unnatural-causes and suicide mortality, with a more pronounced reduction during the pandemic for individuals who self-harmed. Reduction of suicide rates during the pandemic has been reported in other countries (Pirkis et al., 2021). This decrease may be reflected in subsequent mortality of those who self-harmed, a robust risk factor for suicide. Increased unknown-cause mortality during the pandemic may potentially be partially explained by misclassification of suicide deaths (John et al., 2018) and the increased death registration delays due to COVID-19 for deaths, including suicides, which require coroners’ inquests (Office for National Statistics, 2021). Small numbers of deaths reduced statistical power and limited our ability to perform stratified analyses.

Our findings of a widening mortality inequality between individuals who self-harmed and the general population during the COVID-19 pandemic are concerning. Our data do not indicate elevated mortality following self-harm event(s) that occurred during the pandemic. Rather, the self-harm population might be more vulnerable to COVID-19-related adversities (risk of infection and comorbidities, reduced access to care). Timely policies, assessments, crisis pathways, and interventions for at-risk individuals are necessary to ensure those who self-harm receive effective support and to reduce inequalities. We revealed a discernible, albeit nonstatistically significant increase of mortality gap for individuals younger than 65 years compared to 65 years or older. We argue for more targeted interventions aimed at this working population and further research to focus on the at-risk social groups. The decline in self-harm-related contacts to health services during the pandemic found in this study and others may indicate the presence of unmet/unmanaged need that requires prompt attention (DelPozo-Banos et al., 2022). The co-occurrence of a widening mortality gap for individuals who self-harmed and the drop in self-harm-related contacts to health services in the general population during the pandemic needs further investigation. The dynamic nature of the pandemic requires timely data for mitigating relevant risk factors. Large-scale and long-term follow-up studies to monitor the effects of the pandemic on physical and mental health are warranted.

## Electronic Supplementary Material

The electronic supplementary material is available with the online version of the article at https://doi.org/10.1027/0227-5910/a000882

**ESM 1.** Additional tables and figures
**ESM 2.** RECORD statements checklist
**ESM 3.** Detailed descriptions of statistical analysis and the results of the propensity score matching procedure

## Notes

**Publication Ethics** All research conducted has been completed under the permission and approval of the SAIL independent Information Governance Review Panel (IGRP) Project No. 0911.

**Authorship:** All authors were responsible and accountable to all part of works related to the study. AJ conceived the study. AJ, RAL, and AA acquired funding. SCL, MDPB, YF, and AJ contributed to the design of the study. SCL, MDPB, and YF prepared and analyzed the data. SCL, MDPB, YF, and AJ produced the first draft. All authors interpreted the data, contributed to writing and revised the manuscript, and gave the approval to the final version to be published.

**Open Data:** The data used in this study are available in the SAIL Databank at Swansea University, Swansea, the United Kingdom, but as restrictions apply, they are not publicly available. All proposals to use SAIL data are subject to review by an independent Information Governance Review Panel (IGRP). Before any data can be accessed, approval must be given by the IGRP. The IGRP gives careful consideration to each project to ensure proper and appropriate use of SAIL data. When access has been granted, it is gained through a privacy protecting safe haven and remote access system referred to as the SAIL Gateway. SAIL has established an application process to be followed by anyone who would like to access data via SAIL at https://www.saildatabank.com/application-process. Derived data supporting the findings of this study are available from the corresponding author (AJ) on request.
