# Supplementary material for: Widening Excess Mortality During the COVID-19 Pandemic in Individuals Who Self-Harmed: A Whole-Population-Based E-Cohort Study in Wales, UK, April 2016–March 2021
Source: Crisis. 2022 Oct 13;45(2):154–8. doi: 10.1027/0227-5910/a000882 (PMC10999850; doi:10.1027/0227-5910/a000882)
Supplement: Supplementary file 1 [file cri_45_2_154_esm1.pdf]

https://doi.org/10.1027/0227-5910/a000882
© 2022 The Author(s). Distributed as part of a Hogrefe OpenMind article under the license CC BY 4.0 (https://creativecommons.org/licenses/by/4.0)

ESM 1 Table E1: Data sources used in this study.

Database (Abbreviation)	Description	Coverage at time of data extraction*
Welsh Demographic Service (WDSD)	An administrative register of all individuals in Wales that use NHS services, containing anonymised demographics, deaths, and GP practice registration history with anonymized residential data	Up to 08/05/2021
Office for National Statistics (ONS)–Mortality register (ADDD, ADDE)§	Death register of all deaths and causes in Wales, coded using International Classification of Diseases (ICD), version 10 codes, derived from information collected at registration of death. Daily and monthly extracts available.	Up to 06/30/2021
Consolidated Deaths Data Source (CDDS)§	Combination of death records from the Wales Demographic Service Dataset, Master Patient Index, and ONS Deaths.	Up to 08/05/2021
Welsh Longitudinal General Practice (WLGP)	Primary care records with diagnoses, symptoms, investigations, prescribed medication, referrals, coded hospital contacts, and test results coded using Read Codes v2	80% (330/412) of all general practices in Wales up to 06/30/2021
Emergency Department Data Set (EDDS, EDDD)†	Administrative and clinical information (general reason for attendance and attendance group to identify types of contacts) for all NHS Wales Accident and Emergency department attendances.	From 2009 Up to 06/30/2021
Patient Episode Database for Wales (PEDW)‡	Clinical information (specialty and diagnoses) of all NHS Wales hospital admissions (inpatient and day cases) – diagnostic information coded using ICD-10 codes.	Up to 06/30/2021
Outpatient Dataset for Wales (OPDW)	Attendance information for all NHS Wales hospital outpatient appointments, including scheduled outpatient appointments, specialty of care, appointment date and attendance status, and those where the patient failed to attend.	Up to 06/30/2021
Welsh Laboratory Information Systems Database (LIMS)	Clinical system that stores, records and exchanges both COVID-19 antigen/swab tests (pillar 1 and pillar 2), and serology/antibodies tests (pillar 3) results from NHS Wales laboratories.	Form 02/05/2020 to 08/05/2021
Wales Immunisation System (WIS)	Information on vaccination data, priority/risk group information and limited demographic information, e.g., ethnicity, based on all WDSD registered individuals in Wales.	Up to 06/30/2021
Care Homes Data (CARE)	Contains geographic information data about care homes.	Up to 08/05/2021
Education Wales (EDUW)	Schools and Pupil data for Wales, including limited demographics (ethnicity).	School aged pupils in Wales up to 08/05/2021
COVID-19 Shielded People list (CVSP)	List of high-risk individuals advised to self-isolate during COVID-19 pandemic.	Up to 08/05/2021
ONS Census 2011 Wales (CENW)	Data of the latest census held on March 2011 for all people and households.	03/27/2011

* Data have full Wales coverage throughout the study period unless otherwise shown.
§ User guide to mortality statistics - Office for National Statistics. [Online]. Available: https://www.ons.gov.uk/peoplepopulationandcommunity/birthsdeathsandmarriages/deaths/methodologies/userguidetomortalitystatisticsjuly2017
† Digital Health and Care Wales (previously NHS Wales Informatics Service), Emergency Department Data Set structure. [Online]. Available: http://www.datadictionary.wales.nhs.uk/#!WordDocuments/datasetstructure4.htm
‡ Digital Health and Care Wales (previously NHS Wales Informatics Service), Patient Episode Database for Wales Data Set structure. [Online]. Available: http://www.datadictionary.wales.nhs.uk/#!WordDocuments/datasetstructure.htm

https://doi.org/10.1027/0227-5910/a000882
© 2022 The Author(s). Distributed as part of a Hogrefe OpenMind article under the license CC BY 4.0 (https://creativecommons.org/licenses/by/4.0)

ESM 1 Table E2: Variables used in the analysis of risk of mortality in the self-harm cohort.

		Analysis						
		For propensity scores matching (PSM)			For difference-in-difference (DiD) analysis after PSM			
Variable*	Data source**	Included? (Y/N)	Time of Measurement	Categories	Included? (Y/N)	Time-fixed/ varying?	Categories	Time of Measurement
Calendar time	-	N	-	-	Y	Time-varying	Half-year period started from April	-
Self-harm	EDDS, EDDD, PEDW, OPDW, WLGP	N	-	-	Y	Time-fixed	Yes, No	-
Sex	WDSD	Y	-	Male, Female	-	Time-fixed	-	-
Age	WDSD	Y	At index date	Continuous	Y***	Time-varying	10-24, 25-64, 65+	At start dates of each half-year period
Ethnicity	CENW, PEDW, EDUW, WIS	Y	-	White, non-White	N	-	-	-
Area deprivation (WIMD quintile)[1]	WDSD	Y	At index date	Q1 to Q5	N***	-	-	-
Urban/Rural indicator[2]	WDSD	Y	At index date	Urban, Rural	N	-	-	-
Health Board	WDSD	Y	At index date	7 health boards (A-G)	Y	Time-varying	7 health boards	At start dates of each half-year period
Live in care homes[3]	CARE	Y	At index date	Yes, No	Y	Time-varying	Yes, No	At start dates of each half-year period
Ever had COVID-19 infection[4]	LIMS, PEDW, WLGP	N	-	-	Y****	Time-varying	Yes, No	6 months to start dates of each half-year period
In COVID-19 shielded list[5]	CVSP	N	-	-	Y	Time-fixed	Yes, No	-
Charlson Comorbidity Index (unweighted)[6]	PEDW, OPDW, WLGP	Y	1 year before index date to index date	0, 1, 2, 3+	Y	Time-varying	0, 1, 2, 3+	6 months to start dates of each half-year period
Ever Smoked[7]	WLGP	Y	Ever up to index date	Yes, No	N	-	-	-
History of alcohol misuse[8]	EDDS, EDDD, PEDW, OPDW, WLGP	Y	Ever up to index date	Yes, No	Y	Time-varying	Yes, No	At start dates of each half-year period
History of drugs misuse[8]	EDDS, EDDD, PEDW, OPDW, WLGP	Y	Ever up to index date	Yes, No	Y	Time-varying	Yes, No	At start dates of each half-year period
History of depression and anxiety[8]	EDDS, EDDD, PEDW, OPDW, WLGP	Y	Ever up to index date	Yes, No	N	-	-	-
History of severe mental illnesses (SMI)[9]	EDDS, EDDD, PEDW, OPDW, WLGP	Y	Ever up to index date	Yes, No	N	-	-	-
History of developmental disorders[10]	EDDS, EDDD, PEDW, OPDW, WLGP	Y	Ever up to index date	Yes, No	N	-	-	-
History of obsessive compulsory disorder (OCD)/eating disorder[11]	EDDS, EDDD, PEDW, OPDW, WLGP	Y	Ever up to index date	Yes, No	N	-	-	-

https://doi.org/10.1027/0227-5910/a000882
© 2022 The Author(s). Distributed as part of a Hogrefe OpenMind article under the license CC BY 4.0 (https://creativecommons.org/licenses/by/4.0)

Ever prescribed psychotropic medications[12]	WLGP	Y	Ever up to index date	Yes, No	Y	Time-varying	-	6 months to start dates of each half-year period
Ever prescribed opiates medications[12]	WLGP	Y	Ever up to index date	Yes, No	Y	Time-varying	-	6 months to start dates of each half-year period
Number of moves in residential address	WDSD	Y	Ever up to index date	Continuous	N	-	-	-
% of length of residence in Wales to age at index date	WDSD	Y	Ever up to index date	Continuous	N	-	-	-
% of duration of valid GP data length of residence in Wales[13]	WDSD, WLGP	Y	Ever up to index date	Continuous	N	-	-	-

* All references for the list of codes used are cited in the References list in ESM 3.
** Refer to Table E1 in ESM 1 for abbreviations of data sources
*** Subgroup analyses by sex, age, and area deprivation are performed. In those analyses, the corresponding stratified variables are omitted.
**** Modeling for both including and excluding the COVID-19 infection variable is performed.
[1] Welsh Index of Multiple Deprivation with Q5 as the most deprived areas, refer to Welsh Government (2017) for the detailed methodological descriptions.
[2] Refer to Barham and Begum (2006) for the detailed methodological descriptions.
[3] Refer to Hollinghurst et al. (2021) for the detailed methodological descriptions.
[4] Refer to DelPozo-Banos et al. (2021) for the detailed methodological descriptions.
[5] Refer to https://nwis.nhs.wales/news/latest-news/identifying-vulnerable-patient-lists/ and https://nwis.nhs.wales/coronavirus/digital-support-updates-for-healthcare-professionals/identifying-shielding-patients/ for the identification of vulnerable patients.
[6] Refer to Charlson et al. (1987) for its definition and Khan et al. (2010) and for the list of codes used.
[7] Refer to Atkinson et al. (2017) for the detailed methodological descriptions.
[8] Refer to John et al. (2020) for list of codes used.
[9] Refer to John et al. (2018) for list of codes used.
[10] It consists of autism spectrum disorder, attention deficit hyperactivity disorder, learning difficulties, and conduct disorders. Refer to Burt et al. (2004), Brophy et al. (2018), Underwood et al. (2019), and John et al. (2019) for list of codes used.
[11] Refer to John et al. (2021) for list of codes used.
[12] Refer to John et al. (2020) for list of codes used.
[13] Refer to Davies et al. (2018) and Thayer et al. (2020) for the description of defining duration of valid GP data.

https://doi.org/10.1027/0227-5910/a000882
© 2022 The Author(s). Distributed as part of a Hogrefe OpenMind article under the license CC BY 4.0 (https://creativecommons.org/licenses/by/4.0)

ESM 1 Table E3. Sample characteristics of the self-harm cohort before and after propensity score matching (PSM).

		Self-harm (unmatched)		No self-harm (unmatched)		Self-harm (matched)		No self-harm (matched)	
		Numbers	%	Numbers	%	Numbers	%	Numbers	%
Total		45,422	-	2,558,599	-	43,368	-	43,368	-
Sex	Male	20,843	45.9	1,286,132	50.3	19,859	45.8	19,859	45.8
	Female	24,579	54.1	1,272,467	49.7	23,509	54.2	23,509	54.2
Age	10-24 yr	17,342	38.2	492,480	19.2	16,609	38.3	16,609	38.3
	25-64 yr	24,460	53.9	1,469,182	57.4	23,186	53.5	23,186	53.5
	65 yr +	3,620	8.0	596,937	23.3	3,573	8.2	3,573	8.2
Ethnicity	White	40,495	89.2	2,216,190	86.6	38,662	89.1	38,812	89.5
	non-White	1,227	2.7	97,819	3.8	1,205	2.8	1,144	2.6
	Unknown	3,700	8.1	244,590	9.6	3,501	8.1	3,412	7.9
WIMD quintile* (Q5: most deprived)	Q1	5,525	12.2	527,022	20.6	5,323	12.3	5,323	12.3
	Q2	6,293	13.9	512,743	20.0	6,047	13.9	6,047	13.9
	Q3	8,246	18.2	525,543	20.5	7,871	18.1	7,871	18.1
	Q4	11,045	24.3	504,395	19.7	10,518	24.3	10,518	24.3
	Q5	14,313	31.5	488,896	19.1	13,609	31.4	13,609	31.4
Urban/Rural indicator*	Rural	11,204	24.7	795,666	31.1	10,780	24.9	10,848	25.0
	Urban	34,218	75.3	1,762,933	68.9	32,588	75.1	32,520	75.0
Health board*	A	8,264	18.2	567,260	22.2		18.4	7,947	18.3
	B	5,179	11.4	310,042	12.1	4,877	11.2	4,837	11.2
	C	8,366	18.4	316,203	12.4	7,819	18.0	7,984	18.4
	D	5,453	12.0	412,414	16.1	5,316	12.3	5,166	11.9
	E	7,752	17.1	362,412	14.2	7,390	17.0	7,472	17.2
	F	8,821	19.4	483,684	18.9	8,487	19.6	8,420	19.4
	G	1,587	3.5	106,584	4.2	1,518	3.5	1,542	3.6
Live in care homes*		258	0.6	12,570	0.5	236	0.5	224	0.5
Charlson Comorbidity Index** (unweighted)	0	39,088	86.1	2,262,893	88.4	37,480	86.4	37,466	86.4
	1	4,770	10.5	229,909	9.0	4,441	10.2	4,483	10.3
	2	979	2.2	46,254	1.8	902	2.1	911	2.1
	3+	585	1.3	19,543	0.8	545	1.3	508	1.2
Ever smoked***		26,533	58.4	1,197,986	46.8	24,718	57.0	24,804	57.2
History of***	Alcohol misuse	10,355	22.8	126,585	4.9	8,780	20.2	8,521	19.6
	Drugs misuse	7,970	17.5	41,361	1.6	6,337	14.6	6,101	14.1
	Depression and anxiety	26,560	58.5	441,270	17.2	24,618	56.8	24,829	57.3
	SMI	6,238	13.7	73,554	2.9	5,276	12.2	5,214	12.0
	Developmental disorders	4,124	9.1	47,736	1.9	3,472	8.0	3,521	8.1
	OCD/Eating disorders	2,498	5.5	33,915	1.3	2,165	5.0	2,193	5.1
Ever prescribed***	Psychotropics	29,129	64.1	928,050	36.3	27,183	62.7	27,515	63.4
	Opiates	24,018	52.9	1,169,109	45.7	22,637	52.2	22,853	52.7
Number of moves***	0	7,623	16.8	775,127	30.3	7,558	17.4	7,055	16.3
	1	7,375	16.2	612,756	23.9	7,282	16.8	7,426	17.1
	2	6,328	13.9	414,684	16.2	6,212	14.3	6,455	14.9
	3+	24,096	53.0	756,032	29.5	22,316	51.5	22,432	51.7
% of length of residence in Wales to age at index date***	[0-20]	18,687	41.1	813,086	31.8	17,202	39.7	17,428	40.2
	(20-40]	10,752	23.7	773,235	30.2	10,476	24.2	10,115	23.3
	(40-60]	5,603	12.3	472,354	18.5	5,504	12.7	5,682	13.1
	(60-80]	3,174	7.0	163,141	6.4	3,108	7.2	3,073	7.1
	(80-100]	7,206	15.9	336,783	13.2	7,078	16.3	7,070	16.3
% of valid GP data to length of residence in Wales***	[0-20]	6,739	14.8	522,858	20.4	6,484	15.0	6,539	15.1
	(20-40]	1,473	3.2	93,064	3.6	1,411	3.3	1,382	3.2
	(40-60]	1,970	4.3	158,646	6.2	1,904	4.4	1,780	4.1
	(60-80]	2,799	6.2	214,576	8.4	2,703	6.2	2,752	6.3
	(80-100]	32,441	71.4	1,569,455	61.3	30,866	71.2	30,915	71.3
Follow-up period (years)		2.8 (1.5, 4.0)		2.8 (1.6, 4.1)		2.7 (1.5 - 4.0)		2.8 (1.5, 4.0)	

* measured at index dates

** measured a period from 1 year before to index dates

*** measured from available record up to index dates

**** expressed as median and interquartile range

https://doi.org/10.1027/0227-5910/a000882
© 2022 The Author(s). Distributed as part of a Hogrefe OpenMind article under the license CC BY 4.0 (https://creativecommons.org/licenses/by/4.0)

ESM 1 Table E4. Diagnostic checks on PSM of the self-harm cohort by absolute standardized mean differences (SMDs) and variance ratios.

		Unmatched						Matched					
		Self-harm		No-self-harm		Absolute	Variance	Self-harm		No-self-harm		Absolute	Variance
Variable		Mean	Variance	Mean	Variance	SMD	ratio	Mean	Variance	Mean	Variance	SMD	ratio
	Sex	0.541	0.248	0.497	0.250	0.088	0.993	0.542	0.248	0.542	0.248	0.000	1.000
	Age	34.556	333.773	46.444	445.157	0.602	0.750	34.701	341.914	34.793	341.951	0.005	1.000
WIMD quintile, urban/rural indicator (Q5: most deprived)	Q1, Rural	0.032	0.031	0.058	0.054	0.123	0.575	0.033	0.032	0.034	0.032	0.005	0.972
	Q1, Urban	0.089	0.081	0.148	0.126	0.183	0.644	0.090	0.082	0.089	0.081	0.003	1.010
	Q2, Rural	0.059	0.055	0.096	0.087	0.139	0.640	0.060	0.056	0.058	0.055	0.008	1.029
	Q2, Urban	0.080	0.073	0.105	0.094	0.086	0.783	0.080	0.073	0.081	0.075	0.007	0.980
	Q3, Rural	0.064	0.060	0.086	0.079	0.083	0.765	0.065	0.061	0.067	0.063	0.009	0.971
	Q3, Urban	0.117	0.104	0.119	0.105	0.006	0.985	0.117	0.103	0.114	0.101	0.007	1.016
	Q4, Rural	0.056	0.053	0.049	0.047	0.029	1.125	0.056	0.053	0.056	0.053	0.002	1.009
	Q4, Urban	0.187	0.152	0.148	0.126	0.106	1.208	0.186	0.152	0.187	0.152	0.001	0.998
	Q5, Rural	0.035	0.034	0.022	0.021	0.079	1.581	0.035	0.034	0.036	0.035	0.004	0.978
	Q5, Urban	0.280	0.202	0.169	0.141	0.268	1.434	0.279	0.201	0.278	0.201	0.002	1.002
Ethnicity	White	0.893	0.095	0.868	0.115	0.078	0.832	0.891	0.097	0.895	0.094	0.011	1.029
	non-White	0.027	0.026	0.038	0.037	0.063	0.715	0.028	0.027	0.026	0.026	0.009	1.052
	Unknown	0.080	0.073	0.094	0.085	0.050	0.864	0.081	0.074	0.079	0.072	0.008	1.024
Health board	A	0.182	0.149	0.222	0.173	0.099	0.863	0.184	0.150	0.183	0.150	0.001	1.001
	B	0.114	0.101	0.121	0.106	0.022	0.949	0.112	0.100	0.112	0.099	0.003	1.007
	C	0.184	0.150	0.124	0.108	0.169	1.387	0.180	0.148	0.184	0.150	0.010	0.984
	D	0.120	0.106	0.161	0.135	0.119	0.781	0.123	0.108	0.119	0.105	0.011	1.025
	E	0.171	0.142	0.142	0.122	0.080	1.164	0.170	0.141	0.172	0.143	0.005	0.991
	F	0.194	0.156	0.189	0.153	0.013	1.021	0.196	0.157	0.194	0.156	0.004	1.006
	G	0.035	0.034	0.042	0.040	0.035	0.845	0.035	0.034	0.036	0.034	0.003	0.985
Charlson Comorbidity Index (unweighted)	0	0.861	0.120	0.884	0.102	0.072	1.174	0.864	0.117	0.864	0.118	0.001	0.998
	1	0.105	0.094	0.090	0.082	0.051	1.149	0.102	0.092	0.103	0.093	0.003	0.992
	2	0.022	0.021	0.018	0.018	0.025	1.188	0.021	0.020	0.021	0.021	0.001	0.990
	3+	0.013	0.013	0.008	0.008	0.052	1.677	0.013	0.012	0.012	0.012	0.008	1.072
	Live in care homes	0.006	0.006	0.005	0.005	0.011	1.155	0.005	0.005	0.005	0.005	0.004	1.053
History of	Depression and anxiety	0.585	0.243	0.172	0.143	0.939	1.701	0.568	0.245	0.573	0.245	0.010	1.003
	SMI	0.137	0.118	0.029	0.028	0.401	4.243	0.122	0.107	0.120	0.106	0.004	1.010
	Developmental disorders	0.091	0.083	0.019	0.018	0.321	4.508	0.080	0.074	0.081	0.075	0.004	0.987
	OCD/Eating disorders	0.055	0.052	0.013	0.013	0.231	3.974	0.050	0.047	0.051	0.048	0.003	0.988
	Alcohol misuse	0.228	0.176	0.049	0.047	0.535	3.743	0.202	0.161	0.196	0.158	0.015	1.023
	Drugs misuse	0.175	0.145	0.016	0.016	0.562	9.097	0.146	0.125	0.141	0.121	0.016	1.032
Ever prescribed	Psychotropics	0.641	0.230	0.363	0.231	0.580	0.995	0.627	0.234	0.634	0.232	0.016	1.009
	Opiates	0.529	0.249	0.457	0.248	0.144	1.004	0.522	0.250	0.527	0.249	0.010	1.001
	Ever smoked	0.584	0.243	0.468	0.249	0.234	0.976	0.570	0.245	0.572	0.245	0.004	1.001
	Number of moves	3.841	15.413	1.971	5.182	0.583	2.974	3.596	12.874	3.611	12.820	0.004	1.004
	% of length of residence in Wales to age	37.020	986.654	38.295	808.940	0.043	1.220	37.806	990.823	37.786	988.885	0.001	1.002
	% of valid GP data to length of residence	78.350	1,290.295	70.755	1,545.165	0.202	0.835	78.173	1,296.691	78.221	1,294.872	0.001	1.001

https://doi.org/10.1027/0227-5910/a000882
© 2022 The Author(s). Distributed as part of a Hogrefe OpenMind article under the license CC BY 4.0 (https://creativecommons.org/licenses/by/4.0)

ESM 1 Table E5. Summary of ratios of hazard ratios (RHRs) based on DiD analyses for change in all-cause mortality risk during COVID-19 for the matched self-harm cohort.

	COVID-19 infection variable included?	Wave 1			Wave 2		
		RHR	95% CI	p-value	RHR	95% CI	p-value
Main analysis	Yes	2.033	(1.036 - 4.029)	0.042	2.190	(1.117 - 4.287)	0.023
	No	2.043	(1.027 - 4.041)	0.041	2.220	(1.144 - 4.379)	0.021
Robustness check	Yes	1.161	(0.502 - 2.694)	0.723	1.083	(0.717 - 2.520)	0.452
	No	1.144	(0.494 - 2.680)	0.745	1.079	(0.718 - 2.511)	0.453
Sensitivity analysis (using incident self-harm cases)	Yes	1.543	(0.673 - 3.535)	0.306	1.210	(0.508 - 2.871)	0.655
	No	1.539	(0.669 - 3.526)	0.315	1.283	(0.545 - 3.040)	0.564
Sensitivity analysis (analysis without PSM*)	Yes	1.605	(1.112 - 2.321)	0.032	1.617	(1.124 - 2.315)	0.017
	No	1.631	(1.125 - 2.373)	0.030	1.641	(1.141 - 2.376)	0.012

* propensity score matching

https://doi.org/10.1027/0227-5910/a000882
© 2022 The Author(s). Distributed as part of a Hogrefe OpenMind article under the license CC BY 4.0 (https://creativecommons.org/licenses/by/4.0)

ESM 1 Table E6. Summary of RHRs for subgroup analyses for change in all-cause mortality risk during COVID-19 for the matched self-harm cohort.

		Wave 1			Wave 2		
		RHR	95% CI	p-value	RHR	95% CI	p-value
Sex	Male	1.900	(0.792 - 4.572)	0.151	2.646	(1.114 - 6.346)	0.027
	Female	2.841	(0.951 - 8.524)	0.063	1.930	(0.643 - 5.817)	0.243
Ratio of RHR*	(ref: Male)	1.499	(0.366 - 6.104)	0.571	0.727	(0.185 - 2.966)	0.659
Age	< 65 yr	3.291	(0.955 - 11.340)	0.056	3.850	(1.265 - 11.692)	0.018
	65 yr +	1.787	(0.703 - 4.510)	0.217	1.747	(0.707 - 4.277)	0.225
Ratio of RHR*	(ref: 65 yr +)	1.840	(0.394 - 8.622)	0.439	2.205	(0.527 - 9.217)	0.273
WIMD	Q1-Q3	1.546	(0.560 - 4.312)	0.400	2.326	(0.830 - 6.506)	0.106
quinitle	Q4-Q5	2.276	(0.901 - 5.783)	0.083	1.713	(0.674 - 4.364)	0.262
Ratio of RHR*	(ref:Q1-Q3)	1.467	(0.362 - 5.863)	0.589	0.739	(0.189 - 2.973)	0.673

* based on variance-weighted least squares estimation

https://doi.org/10.1027/0227-5910/a000882
© 2022 The Author(s). Distributed as part of a Hogrefe OpenMind article under the license CC BY 4.0 (https://creativecommons.org/licenses/by/4.0)

ESM 1 Table E7a. Numbers of individuals who self-harmed and deaths within the defined periods corresponded to the waves of COVID-19 pandemic outbreak and the respective pre-pandemic/counterfactual periods.

Period*		Date	Self-harm population	Number of deaths	% of death	95% CI
Counterfactual	Pre-C19	Oct 2017 - Mar 2018	5,612	346	6.2	(5.6 - 6.8)
	Wave 1	Apr 2018 - Sep 2018	6,306	369	5.9	(5.3 - 6.5)
	Wave 2	Oct 2018 - Mar 2019	6,199	281	4.5	(4.0 - 5.1)
Actual	Pre-C19	Oct 2019 - Mar 2020	6,105	195	3.2	(2.8 - 3.7)
	Wave 1	Apr 2020 - Sep 2020	5,194	167	3.2	(2.8 - 3.7)
	Wave 2	Oct 2020 - Mar 2021	4,724	125	2.6	(2.2 - 3.2)

* Refer to Methods and Methods in ESM 3 for the definitions of the periods

ESM 1 Table E7b. Summary of difference-in-difference estimators (RORs) of excess mortality for individuals who self-harmed during Wave 1 and Wave 2 of the pandemic.

Wave	Date	ROR*	95% CI	p-value
1	Apr 2020 - Sep 2020	1.064	(0.822 - 1.379)	0.637
2	Oct 2020 - Mar 2021	1.140	(0.862 - 1.507)	0.358

* Ratio of odds ratio. Refer to Methods in ESM 3 for its definition

https://doi.org/10.1027/0227-5910/a000882
© 2022 The Author(s). Distributed as part of a Hogrefe OpenMind article under the license CC BY 4.0 (https://creativecommons.org/licenses/by/4.0)

A

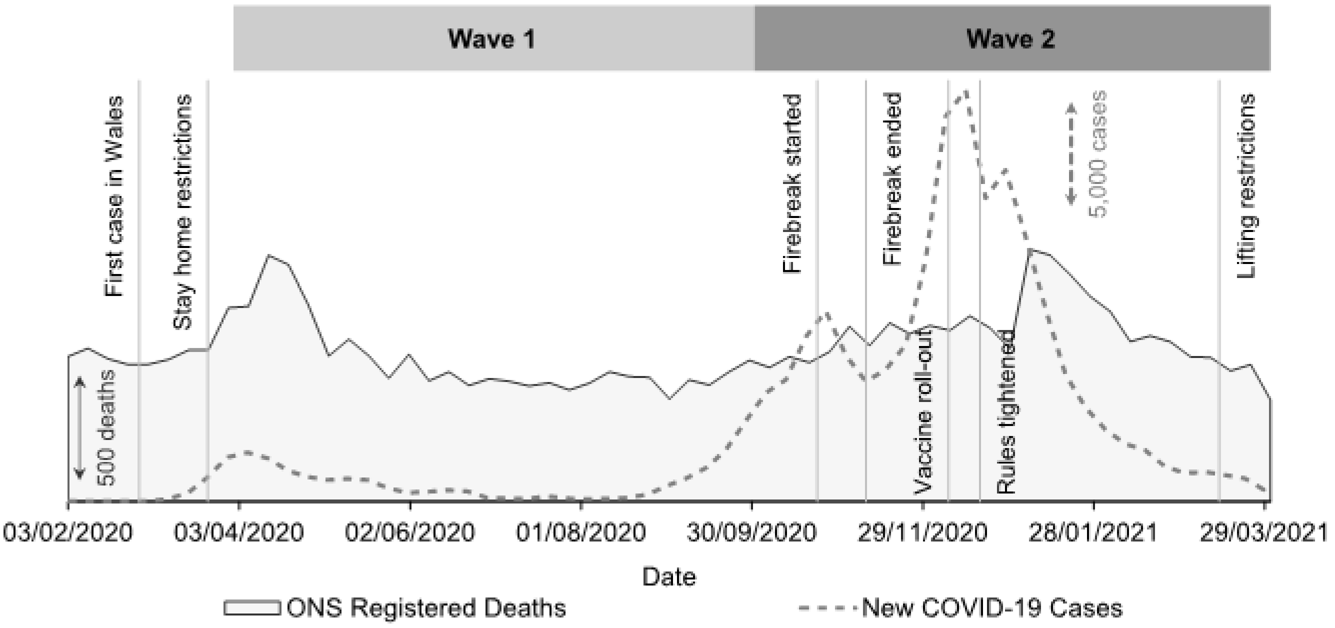

B

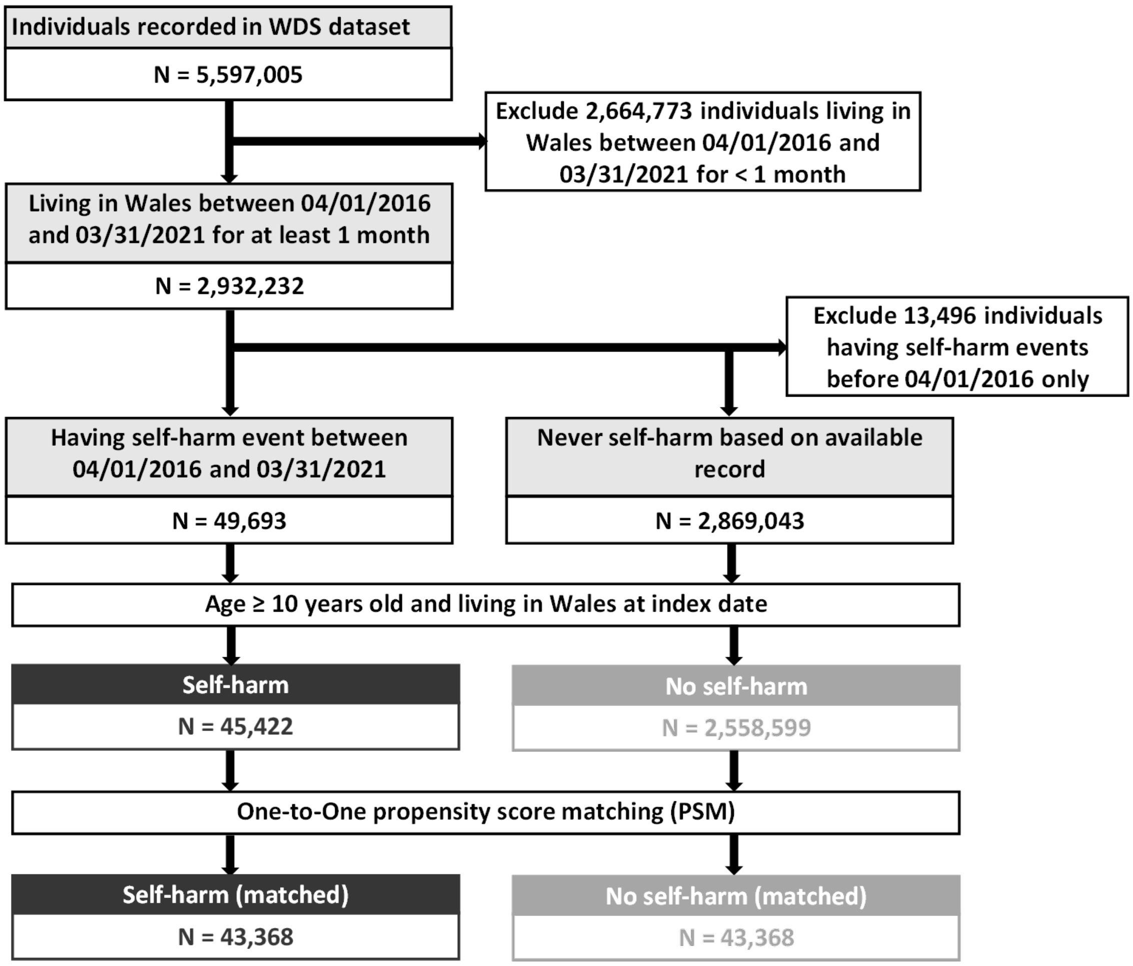

ESM 1 Figure E1. (**A**) The outbreak of the COVID-19 pandemic in Wales, UK from February 2020 to March 2021. Grey dashed line: weekly number of new COVID-19 cases (data extracted from the Public Health Wales COVID-19 interactive data dashboard on 10/25/2021: https://phw.nhs.wales/news/new-novel-coronavirus-covid-19-data-dashboard-launched/). Shaded areas: weekly deaths registered in Wales (data extracted from the Office of National Statistics (ONS) released provisional dataset: https://www.ons.gov.uk/peoplepopulationandcommunity/birthsdeathsandmarriages/deaths/datasets/weeklyprovisionalfiguresondeathsregisteredinenglandandwales). Wave 1 and Wave 2 of the pandemic are defined as from 04/01/2020 to 09/30/2020 and 10/01/2020 to 03/31/2021 respectively in this study. Vertical grey lines: key dates in Wales and the UK in response to COVID-19 (extracted from the Welsh Parliament:

https://doi.org/10.1027/0227-5910/a000882
© 2022 The Author(s). Distributed as part of a Hogrefe OpenMind article under the license CC BY 4.0 (https://creativecommons.org/licenses/by/4.0)
https://research.senedd.wales/research-articles/coronavirus-timeline-welsh-and-uk-governments-response/). 'First case in Wales' (02/28/2020): Wales' first COVID-19 case confirmed; 'Stay home restriction' (03/23/2020): The UK Prime Minister addressed the nation that people were required to stay at home except for very limited purposes; 'Firebreak started' (11/02/2020): The firebreak lockdown in Wales began as national restrictions came into effect; 'Firebreak ended' (11/09/2020): The firebreak lockdown in Wales came to an end; 'Vaccine roll-out' (12/08/2020): Health boards in Wales started administering the COVID-19 vaccine. 'Rules tightened' (12/19/2020): The highest level of restrictions were brought to forward across Wales and restriction rules across UK were tightened. 'Lifting restrictions' (03/13/2021): The stay home restrictions were replaced stay local rule in Wales. (**B**) Study flow diagram.

https://doi.org/10.1027/0227-5910/a000882
© 2022 The Author(s). Distributed as part of a Hogrefe OpenMind article under the license CC BY 4.0 (https://creativecommons.org/licenses/by/4.0)

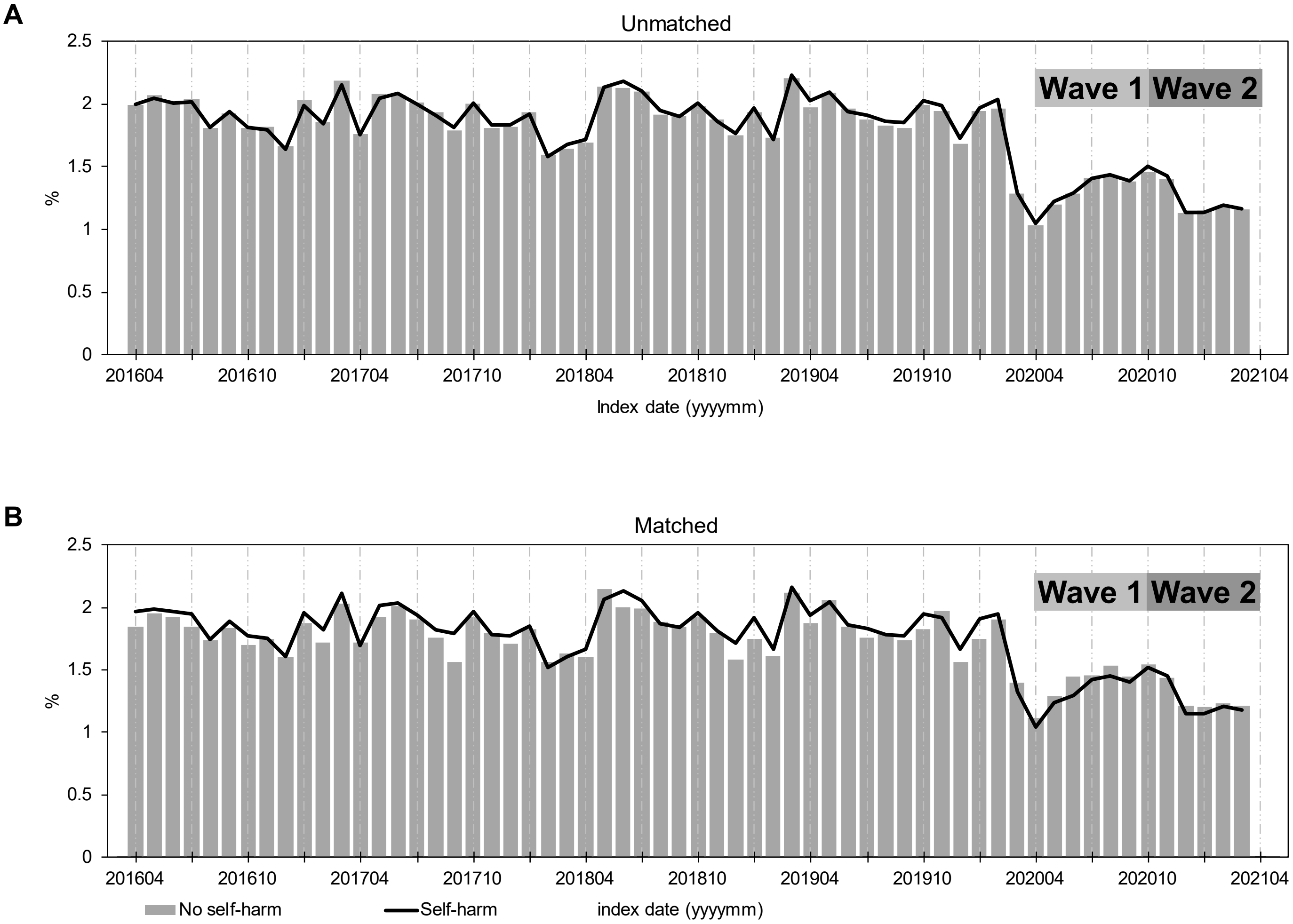

ESM 1 Figure E2. Distribution of index dates of individuals who were ascertained to the ‘self-harm’ and ‘no self-harm’ group before (**A**) and after propensity score matching (**B**). Wave 1’: April 2020-September 2020, ‘Wave 2’: October 2020-March 2021.

https://doi.org/10.1027/0227-5910/a000882
© 2022 The Author(s). Distributed as part of a Hogrefe OpenMind article under the license CC BY 4.0 (https://creativecommons.org/licenses/by/4.0)

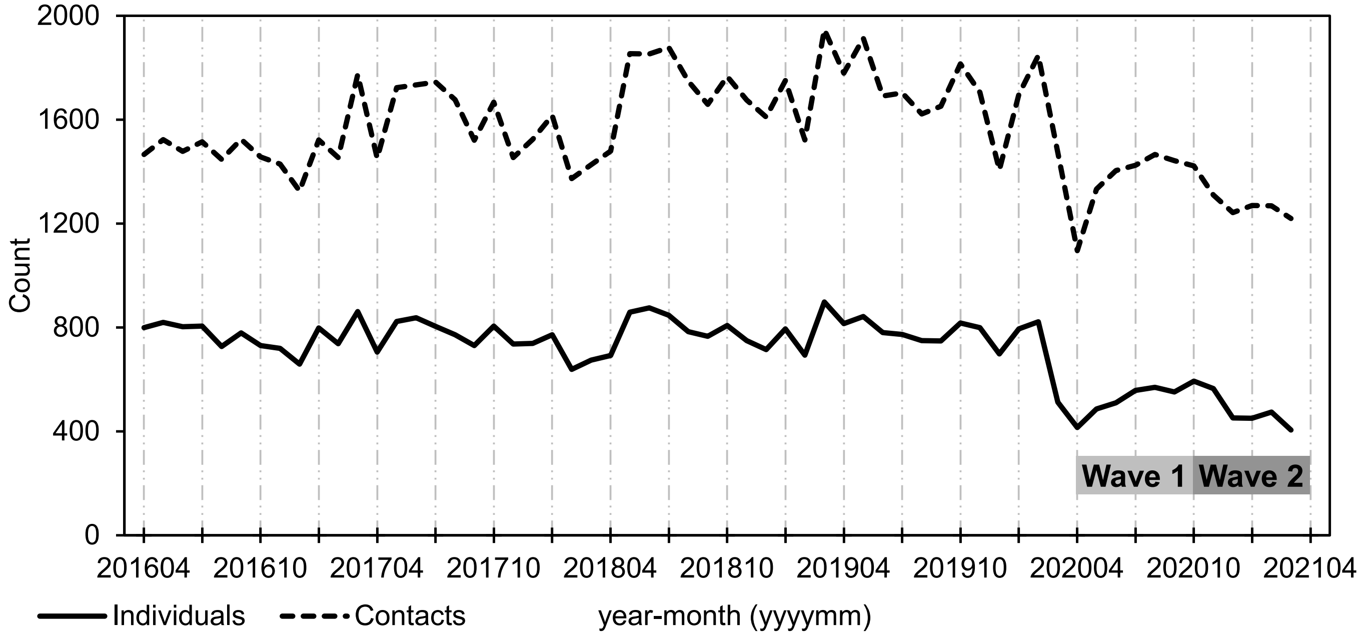

ESM 1 Figure E3. Monthly trend of self-harm (number of individuals and contacts to health services) between April 2016 and March 2021 for the study cohort. 'Wave 1': April 2020-September 2020, 'Wave 2': October 2020-March 2021.

https://doi.org/10.1027/0227-5910/a000882
© 2022 The Author(s). Distributed as part of a Hogrefe OpenMind article under the license CC BY 4.0 (https://creativecommons.org/licenses/by/4.0)

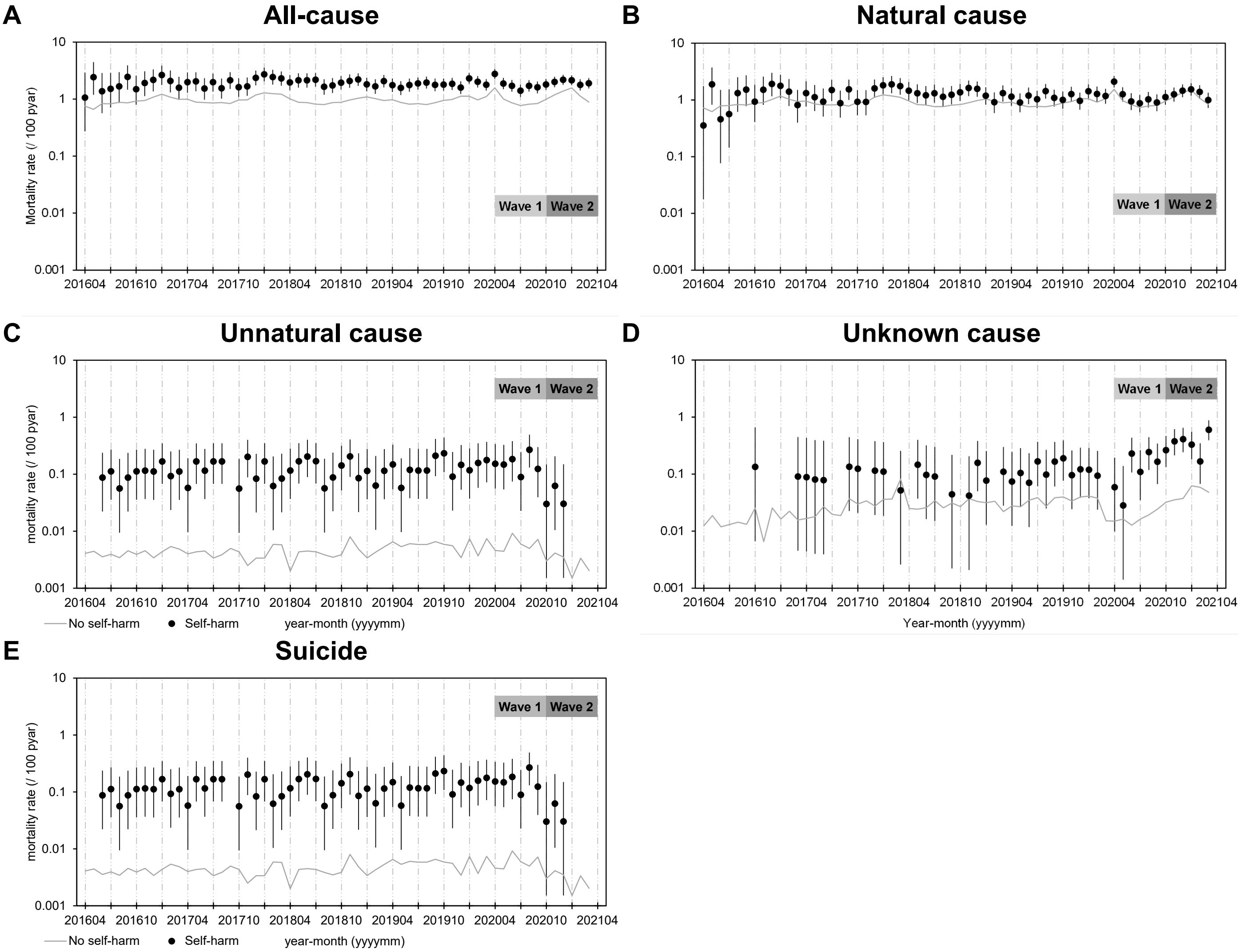

ESM 1 Figure E4. Monthly trends (in log-scale) of all-cause (**A**), natural cause (**B**), unnatural cause (**C**), unknown causes (**D**), and (**E**) suicide crude mortality rates for the 'self-harm' and 'no self-harm' group from the unmatched self-harm cohort between April 2016 and March 2021. Missing data points represent zero death counts. 'Wave 1': April 2020-September 2020, 'Wave 2': October 2020-March 2021. Error bars: 95% CIs. 'pyar': person-years at risk.

https://doi.org/10.1027/0227-5910/a000882
© 2022 The Author(s). Distributed as part of a Hogrefe OpenMind article under the license CC BY 4.0
(https://creativecommons.org/licenses/by/4.0)

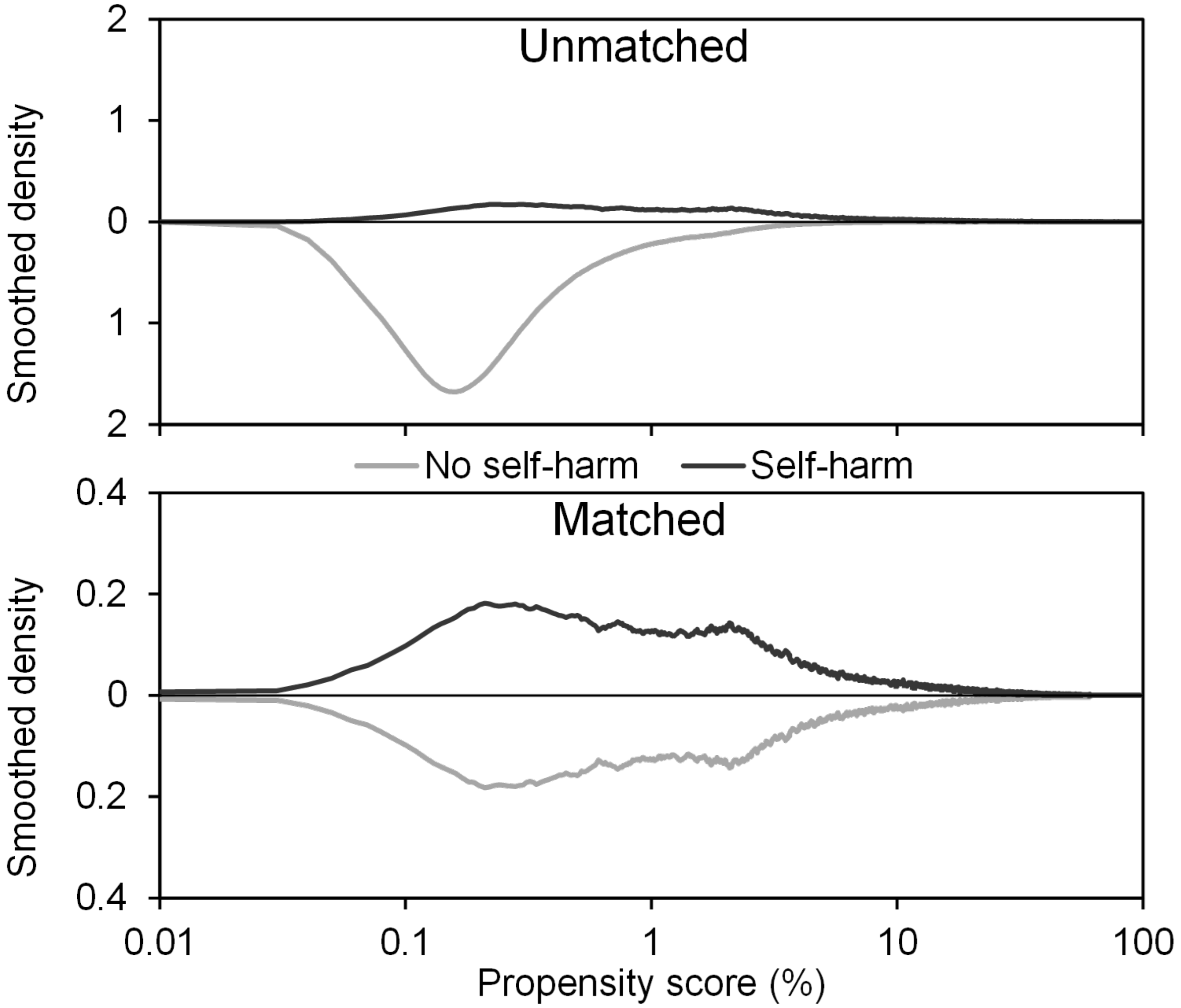

ESM 1 Figure E5. Distribution of propensity score before and after matching of the self-harm cohort. Note the log-scale of the horizontal axes.

https://doi.org/10.1027/0227-5910/a000882
© 2022 The Author(s). Distributed as part of a Hogrefe OpenMind article under the license CC BY 4.0 (https://creativecommons.org/licenses/by/4.0)

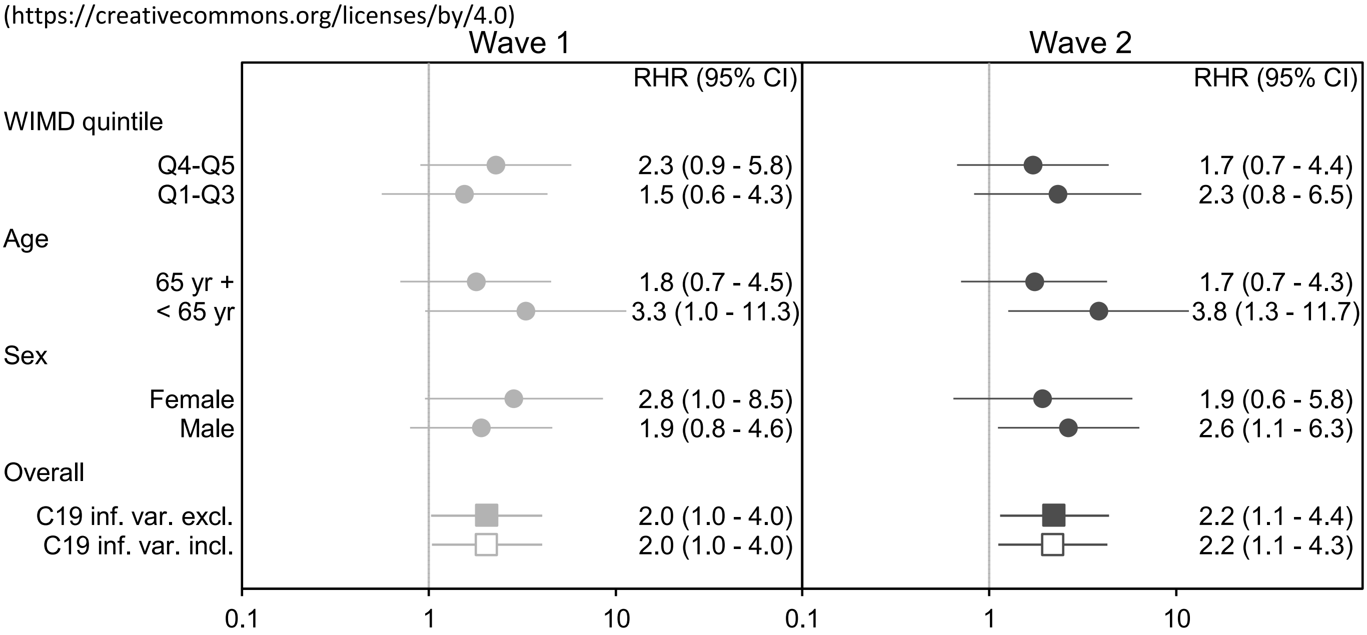

ESM 1 Figure E6. Forest plots of ratios of hazard ratios (RHRs, in log-scale) representing the change in mortality risks during COVID-19 between 'self-harm' and 'no self-harm' group based on difference-in-difference analyses from the matched self-harm cohort. Overall analyses consist of RHRs estimated from models where the COVID-19 infection variable is included ('C19 inf. var. incl.') or excluded ('C19 inf. var. excl.'). Subgroup analyses are performed by sex, age and WIMD quintile ascertained at index date. Error bars: 95% CIs.
