# Supplementary material for: Widening Excess Mortality During the COVID-19 Pandemic in Individuals Who Self-Harmed: A Whole-Population-Based E-Cohort Study in Wales, UK, April 2016–March 2021
Source: Crisis. 2022 Oct 13;45(2):154–8. doi: 10.1027/0227-5910/a000882 (PMC10999850; doi:10.1027/0227-5910/a000882)
Supplement: Supplementary file 2 [file cri_45_2_154_esm2.pdf]

https://doi.org/10.1027/0227-5910/a000882
© 2022 The Author(s). Distributed as part of a Hogrefe OpenMind article under the license CC BY 4.0 (https://creativecommons.org/licenses/by/4.0)

ESM 2. The RECORD statement – checklist of items, extended from the STROBE statement, that should be reported in observational studies using routinely collected health data.

	Item No.	STROBE items	Location in manuscript where items are reported	RECORD items	Location in manuscript where items are reported
**Title and abstract**					
	1	(a) Indicate the study's design with a commonly used term in the title or the abstract (b) Provide in the abstract an informative and balanced summary of what was done and what was found		RECORD 1.1: The type of data used should be specified in the title or abstract. When possible, the name of the databases used should be included.<br><br>RECORD 1.2: If applicable, the geographic region and timeframe within which the study took place should be reported in the title or abstract.<br><br>RECORD 1.3: If linkage between databases was conducted for the study, this should be clearly stated in the title or abstract.	Title and abstract
**Introduction**					
Background rationale	2	Explain the scientific background and rationale for the investigation being reported			Introduction, Figure E1a (ESM 1)
Objectives	3	State specific objectives, including any prespecified hypotheses			Methods
**Methods**					
Study Design	4	Present key elements of study design early in the paper			Methods
Setting	5	Describe the setting, locations, and relevant dates, including			Methods

https://doi.org/10.1027/0227-5910/a000882
© 2022 The Author(s). Distributed as part of a Hogrefe OpenMind article under the license CC BY 4.0 (https://creativecommons.org/licenses/by/4.0)

		periods of recruitment, exposure, follow-up, and data collection			
Participants	6	*(a) Cohort study* - Give the eligibility criteria, and the sources and methods of selection of participants. Describe methods of follow-up<br>*Case-control study* - Give the eligibility criteria, and the sources and methods of case ascertainment and control selection. Give the rationale for the choice of cases and controls<br>*Cross-sectional study* - Give the eligibility criteria, and the sources and methods of selection of participants<br><br>*(b) Cohort study* - For matched studies, give matching criteria and number of exposed and unexposed<br>*Case-control study* - For matched studies, give matching criteria and the number of controls per case		RECORD 6.1: The methods of study population selection (such as codes or algorithms used to identify subjects) should be listed in detail. If this is not possible, an explanation should be provided.<br><br>RECORD 6.2: Any validation studies of the codes or algorithms used to select the population should be referenced. If validation was conducted for this study and not published elsewhere, detailed methods and results should be provided.<br><br>RECORD 6.3: If the study involved linkage of databases, consider use of a flow diagram or other graphical display to demonstrate the data linkage process, including the number of individuals with linked data at each stage.	Methods, Table E1 and Figure E1 (ESM 1)
Variables	7	Clearly define all outcomes, exposures, predictors, potential confounders, and effect modifiers. Give diagnostic criteria, if applicable.		RECORD 7.1: A complete list of codes and algorithms used to classify exposures, outcomes, confounders, and effect modifiers should be provided. If these cannot be reported, an explanation should be provided.	Methods, Table E1, Table E2 (ESM 1), Methods (ESM 3)
Data sources/ measurement	8	For each variable of interest, give sources of data and details of methods of assessment (measurement).			Methods, Table E1, Table E2 (ESM 1)

https://doi.org/10.1027/0227-5910/a000882
© 2022 The Author(s). Distributed as part of a Hogrefe OpenMind article under the license CC BY 4.0 (https://creativecommons.org/licenses/by/4.0)

		Describe comparability of assessment methods if there is more than one group			
Bias	9	Describe any efforts to address potential sources of bias			Methods, Figure E2 (ESM 1), Methods and Results (ESM 3)
Study size	10	Explain how the study size was arrived at			N/A
Quantitative variables	11	Explain how quantitative variables were handled in the analyses. If applicable, describe which groupings were chosen, and why			Methods, Table E2 (ESM 1), Methods (ESM 3)
Statistical methods	12	(a) Describe all statistical methods, including those used to control for confounding<br>(b) Describe any methods used to examine subgroups and interactions<br>(c) Explain how missing data were addressed<br>(d) *Cohort study* - If applicable, explain how loss to follow-up was addressed<br>*Case-control study* - If applicable, explain how matching of cases and controls was addressed<br>*Cross-sectional study* - If applicable, describe analytical methods taking account of sampling strategy<br>(e) Describe any sensitivity analyses			Methods, Table E2 (ESM 1), Methods and Results (ESM 3)

https://doi.org/10.1027/0227-5910/a000882
© 2022 The Author(s). Distributed as part of a Hogrefe OpenMind article under the license CC BY 4.0 (https://creativecommons.org/licenses/by/4.0)

Data access and cleaning methods		..		RECORD 12.1: Authors should describe the extent to which the investigators had access to the database population used to create the study population.<br><br>RECORD 12.2: Authors should provide information on the data cleaning methods used in the study.	Methods, Table E1 (ESM 1)
Linkage		..		RECORD 12.3: State whether the study included person-level, institutional-level, or other data linkage across two or more databases. The methods of linkage and methods of linkage quality evaluation should be provided.	Methods, Table E1 (ESM 1)
**Results**					
Participants	13	(a) Report the numbers of individuals at each stage of the study (*e.g.*, numbers potentially eligible, examined for eligibility, confirmed eligible, included in the study, completing follow-up, and analysed)<br>(b) Give reasons for non-participation at each stage.<br>(c) Consider use of a flow diagram		RECORD 13.1: Describe in detail the selection of the persons included in the study (*i.e.,* study population selection) including filtering based on data quality, data availability and linkage. The selection of included persons can be described in the text and/or by means of the study flow diagram.	Results, Figure E1 (ESM 1)
Descriptive data	14	(a) Give characteristics of study participants (*e.g.*, demographic, clinical, social) and information on exposures and potential confounders<br>(b) Indicate the number of participants with missing data for each variable of interest			Results, Table E3, Table E4 & Figure E1, Figure E2 and Figure E5 (ESM 1)

https://doi.org/10.1027/0227-5910/a000882
© 2022 The Author(s). Distributed as part of a Hogrefe OpenMind article under the license CC BY 4.0 (https://creativecommons.org/licenses/by/4.0)

		(c) *Cohort study* - summarise follow-up time (*e.g.*, average and total amount)			
Outcome data	15	*Cohort study* - Report numbers of outcome events or summary measures over time<br>*Case-control study* - Report numbers in each exposure category, or summary measures of exposure<br>*Cross-sectional study* - Report numbers of outcome events or summary measures			Results, Figure 1, Figure E3, Figure E4 (ESM 1), Results (ESM 3)
Main results	16	(a) Give unadjusted estimates and, if applicable, confounder-adjusted estimates and their precision (e.g., 95% confidence interval). Make clear which confounders were adjusted for and why they were included<br>(b) Report category boundaries when continuous variables were categorized<br>(c) If relevant, consider translating estimates of relative risk into absolute risk for a meaningful time period			Results, Table E5, Figure E6 (ESM 1)
Other analyses	17	Report other analyses done—e.g., analyses of subgroups and interactions, and sensitivity analyses			Results, Table E6, Table E7, Figure E6 (ESM 1)
**Discussion**					
Key results	18	Summarise key results with reference to study objectives			Discussion

https://doi.org/10.1027/0227-5910/a000882
© 2022 The Author(s). Distributed as part of a Hogrefe OpenMind article under the license CC BY 4.0 (https://creativecommons.org/licenses/by/4.0)

Limitations	19	Discuss limitations of the study, taking into account sources of potential bias or imprecision. Discuss both direction and magnitude of any potential bias		RECORD 19.1: Discuss the implications of using data that were not created or collected to answer the specific research question(s). Include discussion of misclassification bias, unmeasured confounding, missing data, and changing eligibility over time, as they pertain to the study being reported.	Discussion
Interpretation	20	Give a cautious overall interpretation of results considering objectives, limitations, multiplicity of analyses, results from similar studies, and other relevant evidence			Discussion
Generalisability	21	Discuss the generalisability (external validity) of the study results			Discussion
**Other Information**					
Funding	22	Give the source of funding and the role of the funders for the present study and, if applicable, for the original study on which the present article is based			Title page
Accessibility of protocol, raw data, and programming code		..		RECORD 22.1: Authors should provide information on how to access any supplemental information such as the study protocol, raw data, or programming code.	Title page

*Reference: Benchimol EI, Smeeth L, Guttmann A, Harron K, Moher D, Petersen I, Sørensen HT, von Elm E, Langan SM, the RECORD Working Committee. The REporting of studies Conducted using Observational Routinely-collected health Data (RECORD) Statement. *PLoS Medicine* 2015; in press.

*Checklist is protected under Creative Commons Attribution (CC BY) license.
