# Supplementary material for: Widening Excess Mortality During the COVID-19 Pandemic in Individuals Who Self-Harmed: A Whole-Population-Based E-Cohort Study in Wales, UK, April 2016–March 2021
Source: Crisis. 2022 Oct 13;45(2):154–8. doi: 10.1027/0227-5910/a000882 (PMC10999850; doi:10.1027/0227-5910/a000882)
Supplement: Supplementary file 3 [file cri_45_2_154_esm3.pdf]

https://doi.org/10.1027/0227-5910/a000882

© 2022 The Author(s). Distributed as part of a Hogrefe OpenMind article under the license CC BY 4.0 (https://creativecommons.org/licenses/by/4.0)

ESM 3. Methods

Data linkage

All data linkage within the Secure Anonymised Information Linkage (SAIL) databank are treated in accordance with the Data Protection Act 2018 and is compliant with the General Data Protection Regulation. Appropriate disclosure control methods were used to restrict the reporting of small numbers (i.e., categories containing less than 10 individuals). Data linkage between database were performed by identity matching and creation of unique anonymized linkage identifier (anonymized linking field) to be linked across datasets. This was conducted via the NHS Wales Informatics Service, a trusted organisation mandated to hold personally identifiable data. Further encryption of datasets using deterministic matching was based on NHS number or probabilistic matching using available demographics (first name, surname, date of birth, gender, and Phonex and Soundex versions of names) based on the Welsh Demographic Service dataset (Ford et al., 2009, Lyons et al., 2009). For probabilistic linkage, a matching score was calculated to reflect the odds of matches of demographic variables for an individual. We included individuals whose data were either deterministically linked or probabilistically linked with matching score of ≥0.9. With the matching criteria, overall accuracies of ≥99.8% could be attained and ≥94.1% of the records could be successfully linked as previously demonstrated (Lyons et al., 2009).

Statistical analysis

We used SQL DB2 to interrogate data within the SAIL Databank. Statistical analyses were performed using Stata 16.1 (StataCorp, 2019). The level of statistical significance was set at $p = 0.05$. All mortality data were expressed as numbers and rates (numbers/person-years at risk).

Propensity score matching (PSM) for the self-harm cohort

To reduce potential biases of baseline characteristics between the 'self-harm' and 'no self-harm' group, we conducted PSM procedure by first obtaining PSs from a multivariable logistic regression with all extracted covariates summarized in Table E2 in ESM 1 as main effects and interactions. Continuous covariates were transformed into restricted cubic splines with five to seven knots (Harrell, 2001). Model fitting was a recurring process guided by Akiake (AIC), Bayesian Information Criteria (BIC) (Akaike, 1974; Schwarz, 1978), and the area under the receiver operating characteristics curve (Hosmer et al., 2013).

One-to-one nearest-neighbor matching without replacement, with a caliper set to be 20% of the standard deviation of the logit transform of the propensity scores with common support (Stuart, 2010) was performed. To facilitate later subgroup analyses of mortality risk by sex, age group, and area deprivation (Welsh Index of Multiple Deprivation, WIMD quintile), we used overall PSs to match within combinations of subgroup strata (two sexes by three age groups by five WIMD groups) and aggregated to create the full matched cohort (Wang et al., 2018). With this approach, only one model was fit and subgroups were fully nested within the main matched cohort.

We performed quality checks on the PSM procedure (Austin & Stuart, 2015), including assessment of balance of covariates before and after matching by 1) comparison of the absolute standardised differences of the covariates and 2) graphical comparison of the

https://doi.org/10.1027/0227-5910/a000882

© 2022 The Author(s). Distributed as part of a Hogrefe OpenMind article under the license CC BY 4.0 (https://creativecommons.org/licenses/by/4.0)

distributions of PSs between two sub-cohorts. Results of PSM and quality checks are summarized in Results in ESM 3, Table E3, Table E4, and Figure E5 in ESM 1.

Modelling for mortality risk and contrast of model coefficients by Difference-in-Difference (DiD) for the self-harm cohort

Overall modeling specifications and postestimations

We quantified the mortality gap between the 'self-harm' and 'no-self-harm' group for the whole study period by fitting multivariable time-stratified Cox proportional hazards regression on the matched self-harm cohort to assess mortality risks of self-harm as time-stratified (half-year) hazard ratios (HRs) over the whole study period (April 2016-March 2021). The main independent variables were calendar time represented by half-year periods begin in April and October each year and indicator of self-harm as main effects and second order interaction. Other covariates listed in Table E2 in ESM 1 were included in the model as main effects and all time-varying covariates were discrete, measured either at the start dates of each time point (t) or within an interval of 6 months up to the start dates of each time point, i.e., [t - 6 months, t) (Table E2 in ESM 1) (Carr et al., 2017). The criteria of including these covariates were based on model fitting statistics (AIC/BIC) and the Harrell's concordance index for goodness of fit of survival models (Harrell et al., 1982) calculated as previously described for models with discrete time-varying covariates (Newson, 2010). All models were stratified by the matched set and the proportional hazards assumption was checked for all covariates based on visual inspection and test of Schoenfeld residuals.

We calculated the DiD estimators to assess if HRs corresponding to the periods of the pandemic outbreak (Wave 1: April 2020-September 2020 and Wave 2: October 2020-March 2021) were higher than HRs from pre-pandemic periods. The first level of difference was the change in HRs corresponded to the pandemic outbreak and the HR corresponded to a defined pre-pandemic period (October 2019-March 2020). The second level of difference was the change in the change in HRs computed from the first level of difference to the values obtained from the respective counterfactual periods (October 2017-March 2019, see Figure 1 for the schematic illustration of the periods). Given the valid common trend assumption, the DiD estimation ensures that changes in HRs before and during the pandemic outbreak are not due to background fluctuations occurred in the counterfactual periods when COVID-19 had not occurred (Dimick & Ryan, 2014; Wing et al., 2018). We reported the results from the DiD estimation as ratios of the hazard ratios (RHRs) of Wave 1 vs. Pre-C19 and Wave 2 vs. Pre-C19 half-year periods in 2020/2021 compared to the respective values from the counterfactual period. RHRs significantly different from one reflect significant difference in change in HRs between Wave 1 (or 2) and the pre-C19 period compared to the same change in HRs in the counterfactual period. All RHRs were compiled using 'contrast' command in Stata after Cox modeling.

Modeling and subgroup analyses

To disentangle the association of COVID-19 infection and all-cause mortality for individuals who self-harmed, we fit two models by including or excluding an indicator variable of COVID-19 infection (Table E2 in ESM 1). We also performed subgroup analyses by sex, age and WIMD and reported sub-group RHRs. We tested the subgroup differences of RHRs using variance-weighted least squares regression (Arya et al., 2013). Due to sample size issues, age and WIMD were regrouped to two categories (for age: <65 years vs. 65+ years; for

https://doi.org/10.1027/0227-5910/a000882

© 2022 The Author(s). Distributed as part of a Hogrefe OpenMind article under the license CC BY 4.0 (https://creativecommons.org/licenses/by/4.0)

WIMD: Q1-Q3 vs. Q4-Q5). Due to model convergence issues, the variable of COVID-19 infection was excluded in all subgroup analyses.

Robustness check on DiD and sensitivity analysis

We conducted a robustness check against the common trend assumption of DiD as previously proposed (Lechner, 2011; Wing et al., 2018). We repeated the RHR estimation by shifting the dates of all Pre-C19, Wave 1 and Wave 2 periods to a year before, i.e., presuming COVID-19 outbreak occurred in the previous year. The common trend assumption was deemed appropriate if RHRs from the robustness check do not significantly different from one.

We conducted sensitivity analysis by repeating the main analysis with a different ascertainment criterion for the ‘self-harm’ group. As opposed to including a mixture of prevalent/incident population of self-harm to the ‘self-harm’ group as in the main analysis, we included only the incident population of self-harm to the ‘self-harm’ group in the sensitivity analysis.

Results of the RHRs from the overall model, alternative model, sub-group analyses, sensitivity analysis, and robustness check are depicted in Figure E6, Table E5, and Table E6 in ESM 1.

Mortality risk for individuals who self-harmed during the COVID-19 pandemic

We compared the mortality risks for individuals who self-harmed during the pandemic (Wave 1 and Wave 2) to those who self-harmed during the pre-pandemic period (pre-C19) by calculating the proportions (%) of mortality for each period. We adopted the DiD approach to take into consideration the proportions of mortality from the respective counterfactual periods (2017-2019) where the pandemic was absent. The DiD estimation was performed using generalised estimating equations using robust variance for parameter estimation (Agresti, 2007). We adopted the binomial distribution with logit link function for proportions. The exchangeable within-subject correlation structure was chosen to circumvent correlation of outcomes over time based on the quasilikelihood under the independence model criterion as described previously (Cui & Qian, 2007; Pan, 2002). The DiD estimator was the interaction term of the dummy variables of ‘Period’ (actual vs. counterfactual) and ‘Wave’ (pre-C19 vs. Wave 1 or Wave 2). We reported mortality proportions for all interested periods and the DiD estimators for Wave 1 and 2 as ratios of odds ratios (RORs) with 95% CIs (Table E7a and Table E7b in ESM 1). RORs >1 reflects an increased mortality risk for individuals who self-harmed during the pandemic over those who self-harmed in the pre-pandemic period.

https://doi.org/10.1027/0227-5910/a000882

© 2022 The Author(s). Distributed as part of a Hogrefe OpenMind article under the license CC BY 4.0 (https://creativecommons.org/licenses/by/4.0)

ESM 3. Results for propensity scores matching (PSM) of the self-harm cohort

We identified 45,422 individuals (1.7% out of 2,604,021) who self-harmed within the study period and met the inclusion criteria (Table E3 and Figure E1b in ESM 1). PSs were created from the best fit logistic model with the area under the receiver operating characteristic curve of 0.862 (95% CI: 0.860-0.863). Distribution of the PSs from the 'self-harm' and 'no-self-harm' group are depicted in Figure E5 in ESM 1. Using one-to-one matching (without replacement) within stratified subgroups of sex, age, and area deprivation, we successfully matched 43,368 individuals from the 'self-harm' group (95.5% out of 45,422) with the same number of individuals from the 'no self-harm' group, whose PSs shared the common support (Table E3 in ESM 1). Distributions of the PSs were very similar between 'self-harm' and 'no self-harm' group after matching (Figure E5 in ESM 1). Diagnostic checks on all used covariates showed that all absolute standardized differences in the matched cohort were substantially lower than those in the unmatched cohort and all variance ratios approached unity after matching (Table E4 in ESM 1), indicating balance of covariates.

https://doi.org/10.1027/0227-5910/a000882

© 2022 The Author(s). Distributed as part of a Hogrefe OpenMind article under the license CC BY 4.0 (https://creativecommons.org/licenses/by/4.0)

References for Methods, Results, Tables, and Figures in ESMs

Agresti, A. (2007). *An Introduction to Categorical Data Analysis* (2nd ed.). John Wiley & Sons Inc.

Akaike, H. (1974). A new look at the statistical model identification. *IEEE Transactions on Automatic Control*, *19*(6), 716–723. https://doi.org/10.1109/TAC.1974.1100705

Arya, M., Li, R., Pegler, K., Sangar, V., Kelly, J. D., Minhas, S., Muneer, A., & Coleman, M. P. (2013). Long-term trends in incidence, survival and mortality of primary penile cancer in England. *Cancer Causes & Control*, *24*(12), 2169–2176. https://doi.org/10.1007/s10552-013-0293-y

Atkinson, M. D., Kennedy, J. I., John, A., Lewis, K. E., Lyons, R. A., Brophy, S. T., Akbari, A., Demmler, J. C., Heaven, M. L., Lacey, A. S., Thayer, D. S., Turner, S. L., & Walters, A. (2017). Development of an algorithm for determining smoking status and behaviour over the life course from UK electronic primary care records. *BMC Medical Informatics and Decision Making*, *17*(1), 2. https://doi.org/10.1186/s12911-016-0400-6

Austin, P. C., & Stuart, E. A. (2015). Moving towards best practice when using inverse probability of treatment weighting (IPTW) using the propensity score to estimate causal treatment effects in observational studies. *Statistics in Medicine*, *34*(28), 3661–3679. https://doi.org/https://doi.org/10.1002/sim.6607

Barham, C., & Begum, N. (2006). The new urban/rural indicator in the Labour Force Survey. *Labour Market Trends*, *114*(12), 409–418.

Brophy, S., Kennedy, J., Fernandez-Gutierrez, F., John, A., Potter, R., Linehan, C., & Kerr, M. (2018). Characteristics of Children Prescribed Antipsychotics: Analysis of Routinely Collected Data. *Journal of Child and Adolescent Psychopharmacology*, *28*(3), 180–191. https://doi.org/10.1089/cap.2017.0003

Burt, A., Maconochie, N., Doyle, P., & Roman, E. (2004). Learning difficulties in children born to male UK nuclear industry employees; analysis from the nuclear industry family study. *Occupational and Environmental Medicine*, *61*(9), 786 LP – 789. https://doi.org/10.1136/oem.2004.012906

Carr, M. J., Ashcroft, D. M., Kontopantelis, E., While, D., Awenat, Y., Cooper, J., Chew-Graham, C., Kapur, N., & Webb, R. T. (2017). Premature Death Among Primary Care Patients With a History of Self-Harm. *The Annals of Family Medicine*, *15*(3), 246–254. https://doi.org/10.1370/AFM.2054

Charlson, M. E., Pompei, P., Ales, K. L., & MacKenzie, C. R. (1987). A new method of classifying prognostic comorbidity in longitudinal studies: development and validation. *Journal of Chronic Diseases*, *40*(5), 373–383. https://doi.org/10.1016/0021-9681(87)90171-8

Cui, J., & Qian, G. (2007). Selection of Working Correlation Structure and Best Model in GEE Analyses of Longitudinal Data. *Communications in Statistics - Simulation and Computation*, *36*(5), 987–996. https://doi.org/10.1080/03610910701539617

Davies, G., Jordan, S., Brooks, C. J., Thayer, D., Storey, M., Morgan, G., Allen, S., Garaiova, I. ., Plummer, S., & Gravenor, M. (2018). Long term extension of a randomised controlled trial of probiotics using electronic health records. *Scientific Reports*, *8*(1), 7668.

DelPozo-Banos, M., Lee, S. C., Friedmann, Y., Akbari, A., Torabi, F., Lloyd, K., Lyons, R. A., & John, A. (2021). Healthcare contacts with self-harm during COVID-19: an e-cohort whole population-based study using individual-level linked routine electronic health

https://doi.org/10.1027/0227-5910/a000882

© 2022 The Author(s). Distributed as part of a Hogrefe OpenMind article under the license CC BY 4.0 (https://creativecommons.org/licenses/by/4.0)

records in Wales, UK, 2016 - March 2021. *PLOS ONE*, accepted.

Dimick, J. B., & Ryan, A. M. (2014). Methods for Evaluating Changes in Health Care Policy: The Difference-in-Differences Approach. *JAMA*, *312*(22), 2401–2402. https://doi.org/10.1001/jama.2014.16153

Ford, D. V., Jones, K. H., Verplancke, J., Lyons, R. A., John, G., Brown, G., Brooks, C. J., Thompson, S., Bodger, O., Couch, T., & Leake, K. (2009). The SAIL Databank: building a national architecture for e-health research and evaluation. *BMC Health Services Research*, *9*(1), 157. https://doi.org/10.1186/1472-6963-9-157

Harrell, F. E. (2001). *Regression Modeling Strategies With applications to linear models, logistic regression, and survival analysis* (1st ed.). Springer-Verlag. https://doi.org/10.1007/978-1-4757-3462-1

Harrell, F. E., Califf, R. M., Pryor, D. B., Lee, K. L., & Rosati, R. A. (1982). Evaluating the Yield of Medical Tests. *JAMA*, *247*(18), 2543–2546. https://doi.org/10.1001/jama.1982.03320430047030

Hollinghurst, J., Lyons, J., Fry, R., Akbari, A., Gravenor, M., Watkins, A., Verity, F., & Lyons, R. A. (2021). The impact of COVID-19 on adjusted mortality risk in care homes for older adults in Wales, UK: a retrospective population-based cohort study for mortality in 2016–2020. *Age and Ageing*, *50*(1), 25–31. https://doi.org/10.1093/ageing/afaa207

Hosmer, D. W., Lemeshow, S., & Sturdivant, R. X. (2013). Applied Logistic Regression: Third Edition. In *Applied Logistic Regression: Third Edition*. wiley. https://doi.org/10.1002/9781118548387

John, A., DelPozo-Banos, M., Gunnell, D., Dennis, M., Scourfield, J., Ford, D. V, Kapur, N., & Lloyd, K. (2020). Contacts with primary and secondary healthcare prior to suicide: case–control whole-population-based study using person-level linked routine data in Wales, UK, 2000–2017. *The British Journal of Psychiatry*, *217*(6), 717–724. https://doi.org/DOI: 10.1192/bjp.2020.137

John, A., Marchant, A., Demmler, J., Tan, J., & DelPozo-Banos, M. (2021). Clinical management and mortality risk in those with eating disorders and self-harm: e-cohort study using the SAIL databank. *BJPsych Open*, *7*(2), e67. https://doi.org/DOI: 10.1192/bjo.2021.23

John, A., McGregor, J., Jones, I., Lee, S. C., Walters, J. T. R., Owen, M. J., O'Donovan, M., DelPozo-Banos, M., Berridge, D., & Lloyd, K. (2018). Premature mortality among people with severe mental illness — New evidence from linked primary care data. *Schizophrenia Research*. https://doi.org/10.1016/j.schres.2018.04.009

John, A., Wood, S., Rees, S., Wang, T., & Marchant, A. (2019). *Mental Healthcare in Young People and Young Adults*. https://www.ncepod.org.uk/2019ypmh.html

Lechner, M. (2011). The Estimation of Causal Effects by Difference-in-Difference Methods. *Foundations and Trends® in Econometrics*, *4*(3), 165–224. https://doi.org/10.1561/0800000014

Lyons, R. a, Jones, K. H., John, G., Brooks, C. J., Verplancke, J.-P., Ford, D. V, Brown, G., & Leake, K. (2009). The SAIL databank: linking multiple health and social care datasets. *BMC Medical Informatics and Decision Making*, *9*, 3. https://doi.org/10.1186/1472-6947-9-3

Newson, R. B. (2010). Comparing the Predictive Powers of Survival Models Using Harrell's C or Somers' D. *The Stata Journal*, *10*(3), 339–358. https://doi.org/10.1177/1536867X1001000303

https://doi.org/10.1027/0227-5910/a000882

© 2022 The Author(s). Distributed as part of a Hogrefe OpenMind article under the license CC BY 4.0 (https://creativecommons.org/licenses/by/4.0)

Pan, W. (2002). Goodness-of-fit Tests for GEE with Correlated Binary Data. *Scandinavian Journal of Statistics*, *29*(1), 101–110. https://doi.org/https://doi.org/10.1111/1467-9469.00091

Schwarz, G. (1978). Estimating the Dimension of a Model. *The Annals of Statistics*, *6*(2), 461–464. https://doi.org/10.1214/aos/1176344136

StataCorp. (2019). *Stata Statistical Software: Release 16*. StataCorp LLC.

Stuart, E. A. (2010). Matching Methods for Causal Inference: A Review and a Look Forward. *Statistical Science*, *25*(1), 1–21. https://doi.org/10.1214/09-STS313

Thayer, D., Rees, A., Kennedy, J., Collins, H., Harris, D., Halcox, J., Ruschetti, L., Noyce, R., & Brooks, C. (2020). Measuring follow-up time in routinely-collected health datasets: Challenges and solutions. *PLOS ONE*, *15*(2), e0228545. https://doi.org/10.1371/journal.pone.0228545

Underwood, J. F. G., Kendall, K. M., Berrett, J., Lewis, C., Anney, R., van den Bree, M. B. M., & Hall, J. (2019). Autism spectrum disorder diagnosis in adults: phenotype and genotype findings from a clinically derived cohort. *British Journal of Psychiatry*, *215*(5), 647–653. https://doi.org/DOI: 10.1192/bjp.2019.30

Wang, S. V, Jin, Y., Fireman, B., Gruber, S., He, M., Wyss, R., Shin, H., Ma, Y., Keeton, S., Karami, S., Major, J. M., Schneeweiss, S., & Gagne, J. J. (2018). Relative Performance of Propensity Score Matching Strategies for Subgroup Analyses. *American Journal of Epidemiology*, *187*(8), 1799–1807. https://doi.org/10.1093/aje/kwy049

Welsh Government. (2017). *Welsh Index of Multiple Deprivation (WIMD) 2014 Revised*. https://gov.wales/docs/statistics/2015/150812-wimd-2014-revised-en.pdf

Wing, C., Simon, K., & Bello-Gomez, R. A. (2018). Designing Difference in Difference Studies: Best Practices for Public Health Policy Research. *Annual Review of Public Health*, *39*(1), 453–469. https://doi.org/10.1146/annurev-publhealth-040617-013507
